# Prioritization of chemical scaffolds using the TDR Targets database: An integrative workflow for *Trypanosoma cruzi* drug discovery

**DOI:** 10.1371/journal.pntd.0014648

**Published:** 2026-08-24

**Authors:** Lionel Urán Landaburu, Mercedes Didier Garnham, Emir Salas Sarduy, Fernán Agüero

**Affiliations:** 1 Escuela de Bio y Nanotecnologías (EByN), Universidad Nacional de San Martín (UNSAM), San Martín, Buenos Aires, Argentina; 2 Instituto de Investigaciones Biotecnológicas (IIBIO), Consejo Nacional de Investigaciones Científicas y Técnicas (CONICET), San Martín, Buenos Aires, Argentina; Tulane University, UNITED STATES OF AMERICA

## Abstract

Chagas disease, caused by the parasite *Trypanosoma cruzi*, faces a critical innovation gap in drug development, with current treatments hindered by toxicity and limited efficacy. To address this, we implemented an integrative chemogenomic workflow using the TDR Targets database to prioritize drug candidates. To prioritize repurposing candidates for *T. cruzi*, we designed a query to retrieve compounds active against validated targets in other organisms, provided an orthologous gene exists in *T. cruzi* and the compound has no recorded activity against trypanosomatids and their associations predicted by the TDR Targets multilayer network. On those associations we applied sequential filters based on metabolic relevance, and commercial availability via the MolPort API obtaining a focused set of 378 high-priority compounds. A central feature of this workflow was the partitioning of these compounds into 16 distinct chemical libraries, each defined by unique scaffolds such as benzamidines, sulfonamides, and azoles. For experimental validation, we manually curated two of these libraries, containing piperazine and nitro derivatives. From the 21 compounds acquired for *in vitro* testing against *T. cruzi* in intracellular models of infection, 8 demonstrated selective trypanocidal activity after first round, with one lead hit achieving a submicromolar EC_50_ value. Crucially, while our experimental focus was on these two series, the remaining 14 curated libraries, representing a broad range of chemical space and putative target associations are fully available for public exploration and further biological assaying. These results demonstrate the efficiency of our prioritization pipeline and provide the scientific community with a pre-filtered, commercially accessible resource to accelerate the discovery of new leads for Chagas disease.

## Author summary

Chagas disease, caused by the parasite *Trypanosoma cruzi*, is a neglected tropical disease with limited treatment options. To accelerate drug discovery, we developed an integrative computational workflow using the TDR Targets database as a starting point to prioritize compounds for repurposing. We retrieved compounds active against validated targets in other organisms, ensuring they had no recorded activity against trypanosomatids and that their associations were predicted by the TDR Targets multilayer network. Based on this, we provide a 16 chemical libraries with 378 compounds for furhter experimental validation. In this work we focused on two of these sublibraries for experimental validation, and from 21 compounds tested, 8 showed selective activity against the parasite. The remaining curated chemical libraries, represent a broad range of chemical space and putative target associations, and are fully available for exploration and further biological assaying. This study demonstrates the efficiency of our prioritization pipeline and provides a valuable resource to accelerate the discovery of new leads for Chagas disease.

## Introduction

The World Health Organization (WHO) has identified 20 Neglected Tropical Diseases (NTDs), which have historically affected impoverished populations across Africa, Asia, and the Americas [1–3]. Among them, Chagas disease represents a major social, economic and public health burden in Latin America and is one of the most prevalent NTDs [4]. Chagas disease is caused by the protozoan parasite *Trypanosoma cruzi*. It is endemic throughout the American continent, affecting approximately 8 million people [5], with an increasing number of cases reported in North America [6]. Clinically, the disease manifests in two distinct phases: a short, mostly asymptomatic acute phase and a decades-long chronic phase, associated with severe complications such as megacolon and potentially fatal cardiomyopathies [7].

Currently, there is no available vaccine for Chagas disease and existing treatment options rely on Nifurtimox (NFX) and Benznidazole (BNZ), two drugs with limited efficacy in chronic infections and considerable toxicity [8]. Thus, the development of new, effective, and safer therapeutic alternatives remains a pressing need. However, the limited commercial incentive for pharmaceutical companies to invest in treatments for NTDs, primarily due to low expected financial returns, has resulted in a striking innovation gap: only 1% of new drugs approved between 1977 and 2002 target these diseases, and none of them for Chagas disease [9,10]. From 2000 to 2011, neglected diseases received just 4% of all new products and 1% of new chemical entities [11]. Although between 2005 and 2024 fifteen new drugs were approved for some neglected tropical diseases, none of these was a new chemical entity for Chagas disease [12,13].

Given the high costs and extended timelines required for *de novo* drug discovery, such strategies have historically been limited for NTDs [14]. Addressing this gap requires the systematic integration of heterogeneous biological and chemical data, a field known as chemogenomics. While global drug repurposing efforts have identified promising compounds for NTDs [15], the available pathogen-specific data remains critically sparse compared to the vast resources for model organisms [2]. This scarcity hence requires data from well-characterized model organisms, particularly for core cellular processes that are evolutionarily conserved. Dedicated bioinformatics strategies are essential for this cross-species integration. Resources like the TDR Targets database [16–19] enable the ranking of potential drug targets and the identification of bioactive compounds by systematically integrating genomic data from pathogens with functional annotations and chemical bioactivity data from model systems [2,3]. This interconnected architecture is particularly suited for NTDs, as it provides a robust framework to leverage bioactivity data from extensively studied organisms to guide drug discovery in pathogens that typically lack high-volume, experimental chemogenomic datasets.

The TDR Targets database integrates a chemogenomics network that is built on three primary entity data types [18,19]: bioactive chemical compounds, protein targets from pathogen and model organism proteomes, and functional annotation nodes (e.g., Pfam domains [20], OrthoMCL ortholog groups [21], KEGG pathways [22]). Distinct layers encode specific relationship types: a compound-compound layer based on structural similarity (Tanimoto coefficient), a protein-annotation affiliation layer that groups targets based on shared annotations, and crucially, a compound-protein bioactivity layer populated by manually curated and literature-derived interactions [18]. This interconnected structure formalizes the chemogenomic space, allowing for graph-based analyses and inference. A key analytical output derived from this network is the Network Druggability Score (NDS), a metric designed to prioritize protein targets that can be associated with known compounds with higher inference confidence. The NDS algorithm evaluates the local topology surrounding each target node and assigns relevance weights to shared annotation nodes based on the statistical over-representation of known druggable (compound-linked) proteins within those functional categories [19]. A target’s NDS, which ranges from 0 to 1, is thus a weighted composite score reflecting the density and confidence of connections to bioactive compounds via informative functional annotations. A high NDS indicates not only that a target resides in a network region enriched with successful pharmacological interactions, but more importantly, that it can be prioritized with greater confidence for compound assignment. This provides a data-driven, multi-evidence proxy for druggability that extends beyond simple sequence similarity.

For instance, an independent study used the TDR Targets database to derive druggability indices that guided a proteome-wide drug repositioning screen against *Plasmodium falciparum*, ultimately predicting 133 approved drugs as candidates against 34 parasite proteins [23]. Similarly, researchers studying drug-resistant *Mycobacterium tuberculosis* combined the TDR Targets database with metabolic pathway analysis to prioritize putative drug targets among resistance-associated genes [24].

While these computational frameworks are robust, a significant challenge remains in translating an abstract chemogenomic graph (often containing millions of bioactivity records) into tangible, testable hypotheses. In this study, we explicitly leverage the multilayer framework of TDR Targets as the foundational step in a novel drug discovery pipeline for *T. cruzi*.

When the multilayered network was first generated [18], the NDS score was used to perform a prioritization of the best ten targets within the ‘TriTryp’ kinetoplastid parasites (*Trypanosoma cruzi*, *T. brucei*, and *Leishmania major*) [18]. Furthermore, other groups have used the TDR Targets-network based engine to guide prioritization of targets and compounds [25–36].

The TDR Targets platform underwent a significant expansion with the release of TDR Targets version 6, which integrated 22 new genomes and extensive updates to chemical and bioactivity data [19]. This version enabled a new ranking of complete proteomes, including *T. cruzi*, based on the NDS. This also led to the definition of 5 Druggability Groups (DGs) or Tiers for targets, with targets classified in group 5 (DG5) representing the highest scores and greatest confidence, and DG1 the lowest. While the foundational work demonstrated the model’s robustness by identifying established targets [19], these prioritizations served primarily as a proof-of-concept and remained without prospective experimental validation.

In this work, we moved beyond *in silico* prioritization to experimental validation. Using the TDR Targets chemogenomic framework, we selected high-confidence *T. cruzi* targets and retrieved all associated bioactive compounds—roughly 180,000 molecules. Through sequential filtering based on metabolic relevance and commercial availability, we narrowed these to 378 candidates representing diverse chemical scaffolds. From 2 selected libraries, we tested 21 compounds *in vitro* and identified 5 with selective trypanocidal activity and low cytotoxicity. This demonstrates that network-based predictions can deliver novel, biologically active compounds, providing a generalizable strategy for drug discovery in neglected diseases.

## Materials and methods

### Ethical statement

All protocols conducted with animals were designed and carried out in accordance with international ethical standards for animal experimentation and were approved by the Institutional Committee for the Care and Use of Experimental Animals of the University of San Martin (CICUAE, protocol numbers: 10/2019 and 28/2024).

### Construction of the screening libraries

The construction of the libraries was carried out in two stages. First, several datasets were collected from multiple sources, with TDR Targets being one of the most relevant. This step was designated as ‘Step 0’. The complete list of datasets is available in Table 1.

**Table 1:** List of datasets collected for generation of the screening libraries.

Source	Dataset	Label	Approx. Size	Type^a^
TDR Targets	NDPI^b^	Compounds obtained by transforming a list of targets into a list of compounds	180 023	(C)
TDR Targets	Tested	Compounds tested (active, inactive, inconclusive) against trypanosomatids	6 619	(C)
DrugBank	Drugbank	FDA-approved drugs, marketed and withdrawn	11 172	(C)
Molport	Available	Commercial availability	> 5 × 10^6^	(C)
Various^c^	OPI^d^	Manually curated high-throughput screening hits against *T. cruzi* not yet included in TDR Targets	36	(C)
TDR Targets	TDR-Targets-D	*T. cruzi* proteins with druggability group ≥ 4	327	(P)
TDR Targets	TDR-Targets-EA	*T. cruzi* proteins involved in energy and amino acid metabolism	44	(P)

a Dataset type: (C) compound library; (P) protein target set.

b network-derived putative interactions.

c Manually compiled dataset from phenotypic screenings [37,38].

d Other putative interactions.

Once collected, the datasets were sequentially combined in order to reduce the number of entities to a manageable size. Initially, in Step 1, all records from the full list of network-derived putative interactions whose compounds were also found in the tested compounds list were removed. The resulting dataset was intersected in Step 2 with the commercially available compounds dataset (available), eliminating all records involving compounds that could not be commercially sourced, thereby generating a new dataset. This was then combined with the DrugBank dataset in an attempt to obtain a quick list of repositionable drugs; however, no intersecting candidates were identified.

Putative targets were also used to reduce the size of the original dataset. To do this, the dataset resulting from the previous operations was intersected in Step 3 with a curated list of genes of interest, both highly druggable (TDR-Targets-D) and presumably involved in energy and amino acid metabolism (TDR-Targets-EA) [39].

Finally, Step 4 comprised structural clustering and outlier removal to select a reduced number of chemical leads. Using MACCS keys and an all-vs-all Tanimoto similarity matrix, we implemented a hierarchical single-linkage clustering approach. A distance (or height) threshold of 0.8 was selected to generate coherent microclusters while preventing the conflation of distinct scaffolds. To mitigate pharmacological promiscuity, we performed a noise removal step by discarding compounds linked to more than 10 different target families, a strategy analogous to PAINS analysis [40,41].

The compound library was subdivided into subsets of molecules containing a particular functional group or core scaffold. Each scaffold was selected because it has been reported in compounds with trypanocidal activity. The scaffolds are listed in Table 2, together with the reference where each scaffold was shown to inhibit parasite growth. For experimental validation, two of these libraries were selected for compound refinement, resulting in a library of 31 piperazine derivatives and another enclosing 16 nitro-containing compounds. From this total of 47 compounds, 21 were commercially acquired for *in vitro* assays.

**Table 2:** Scaffold-based compound libraries.

Scaffold	Library Size	SMILES	Representative Publication	Ref
Azole	91	N1C=CC=C1, N1C=CN=N1, C1N=CC=N1, N1C=CN=C1, N1C=NC=N1	Goad et al (1989)	[42]
Thiazole	49	S1C=CN=C1, S1C=CC=C1	De Oliveira Filho et al (2017)	[43]
Sulfonamide	40	NS(=O)(=O)C1=CC=CC=C1	Chohan et al (2013)	[44]
Benzamidine	37	NC(=O)C1=CC=CC=C1	Vanden Eynde et al (2016)	[45]
Piperazine	31	C1CNCCN1	Ciammaichella et al (2020)	[46]
Morpholine	30	C1COCCN1	Kuettel et al (2007)	[47]
Oxazole	19	O1C=CN=C1, O1C=CC=C1	Rocha et al (2022)	[48]
Nitro	16	O=N(=O)C1=CC=CC=C1	Patterson et al (2014)	[49]
Benzothiazole	16	S1C=NC2=C1C=CC=C2	Racane et al (2021)	[50]
Pyrrolone	11	O=C1NCC=C1	Bethencourt-Estrella et al (2024)	[51]
Chromene	9	C1C=COC2=C1C=CC=C2, O=C1C=COC2=C1C=CC=C2	Batista Jr et al (2008)	[52]
Adenine	8	NC1=NC=NC2=C1NC=N2	Nakajima-Shimada et al (1996)	[53]
Indole	7	N1C=CC2=C1C=CC=C2	Mezencev et al (2007)	[54]
Napthalene Diimide	2	O=C1NC(=O)C2=C3C(C=CC=C13)=CC=C2	Zuffo et al (2019)	[55]
Furan	1	O1C=CC=C1	Zuma et al (2017)	[56]
Thiazolidine	1	O=C1CSC(=S)N1	Moreira et al (2014)	[57]

The Python code used to construct and subdivide the libraries, together with the resulting scaffold-specific subsets, is available on GitHub as a Jupyter notebook.

### Chemical compounds and reagents

Benznidazole (Abarax) powder was a gift from Laboratorios Elea, Argentina. The stock solution was prepared in dimethyl sulfoxide (DMSO) at 20 mM. Other investigational compounds were sourced from the following suppliers through Molport (Riga, Latvia) and Hit2Lead (ChemBridge Corporation, CA, USA) (S2 Table). These compounds were solids and some required solubilization in a small amount of DMSO before dilution in aqueous media.

### Cytotoxicity determination

To determine cytotoxicity, a resazurin (RZ) assay was performed using the same compound concentrations on non-infected Vero cell cultures [58]. Cells were seeded in 96-well plates at a density of 5 × 10^3^ cells/well and cultured for 48 hours. They were then carefully washed with PBS, fed with fresh RPMI medium supplemented with each compound (20 μM), and incubated for 72 hours. Afterward, 10 μL of freshly prepared 10X (440 μM) RZ solution was added to each well. The assay included BNZ (20 μM) and DMSO (0.5%) as controls, along with an untreated cell control and a no-cell control to establish the maximum and minimum reagent reduction readings. In addition, a 100% reduced RZ control (1X RZ solution sterilized by autoclaving in RPMI medium) was included on the plate as a reference to determine when to take the final reduction reading: absorbance was measured at 600 nm every hour until the untreated cell control reached ~ 100% of the reduced RZ control reading. The final measurement was taken after ~ 7 hours of incubation, recording fluorescence intensity with excitation at 535 nm and emission at 595 nm. All measurements were performed using a FilterMax F5 Multimode Microplate Reader (Molecular Devices, CA, USA). Cytotoxicity was measured as a function of production of resorufin (reduction product of resazurin), using Equation 1, where F is fluorescence measurement and μC+ represents the mean of vehicle controls. Compounds and controls were tested in duplicate.


$$\begin{array}{c} \%\;Cell\;viability=\frac{F}{\mu C+}*100 \end{array}$$ (1)

Compounds maintaining ≥ 35% Vero cell viability at the screening concentration were considered for subsequent CC_50_ evaluation based on their *T. cruzi* growth inhibition profiles. For CC_50_ determination, dose–response cytotoxicity assays were performed under the same experimental conditions described above, using at least eight two-fold serial dilutions of each compound. The highest concentrations assayed were 100 μM for CID-5, 50 μM for CID-6 and CID-19, 5 μM for CID-7, and 7.5 μM for CID-21. Each condition was assayed with two technical replicates and between one and three biological replicates. Data were imported into GraphPad Prism version 8.0.1 (GraphPad Software LLC) and analyzed using nonlinear regression to generate log(dose)-response curves with a variable slope. CC_50_values, defined as the concentration causing a 50% reduction in cell viability, were calculated independently for each compound.

### Parasite culture

*T. cruzi* Tulahuen strain parasites constitutively expressing *E. coli* β-galactosidase (Tul β-gal) were a generous gift of Frederick Buckner (University of Washington, USA). Initial stocks of infectious trypomastigotes were obtained through serial passages in CF1 mice. At the peak of parasitemia, transgenic Tul β-gal parasites were obtained from anticoagulated blood of infected mice. For purification, on the same day as the mouse bleeding, the blood sample was diluted with 1 or 2 volumes of PBS and centrifuged at 300 g for 5 minutes; it was then incubated at 37 °C for 40 minutes to promote swimming of the trypomastigotes. This centrifugation and incubation (swimming) step was repeated a second time. After 40 minutes, the supernatant was again collected and centrifuged at 4,500 g for 7–10 minutes to pellet the swimming trypomastigotes. The parasite pellet was washed repeatedly with RPMI medium, with centrifugation between each wash. Before the final wash, an aliquot was taken to count and assess parasite viability.

For all infection assays, cell-derived Tul β-gal trypomastigotes were propagated in Vero cells maintained at 37 °C with 5% CO_2_ in a humidified incubator. Cells were cultured in minimum essential medium (MEM; Gibco Life Technologies) supplemented with 10% fetal bovine serum, 100 U/mL penicillin, and 10 μg/mL streptomycin. Trypomastigotes were collected from culture supernatants 72 hours post-infection by centrifugation at 5,000 x g for 10 minutes. Parasites were routinely maintained in Vero cells for up to 40 passages without detectable loss of β-galactosidase activity. Thereafter, new cultures were initiated from cryopreserved bloodstream trypomastigotes.

### Trypanocidal activity determination

To determine the trypanocidal activity of the investigational compounds, Tul β-gal parasites were used. The enzymatic activity of the β-galactosidase (β-gal) reporter enzyme was used as a proxy for parasite growth and measured via a colorimetric end-point assay using chlorophenol red-β-D-galactopyranoside (CPRG) as substrate [59]. For this assay, $5\times 10^{4}$ trypomastigotes per well were used to infect Vero cells previously seeded (24 h before) in a 96-well plate at a density of $5\times 10^{3}$ cells per well. After 24 hours of infection, cultures were gently washed with PBS to remove free trypomastigotes and then incubated in phenol red-free RPMI medium (Gibco Cat No. 11835030) supplemented with the compound (20 μM). Benznidazole (BNZ), also at 20 μM, was used as a positive control, along with DMSO (0.5%) as a negative/vehicle control, and untreated infected and uninfected controls to establish maximum and minimum β-gal activity readings, respectively.

After incubating the culture with the compound (20 μM, 2 μM, or 0.2 μM) for 72 hours, 100 μL of a freshly prepared solution containing 1% nonyl phenoxypolyethoxylethanol (NP-40) and 100 μM CPRG (Roche Cat No. 10884308001) was added to each well, reaching final concentrations of 0.5% NP-40 and 50 μM CPRG per well. The plates were then incubated for 4 hours at 37 °C, protected from light throughout the incubation. Finally, β-gal activity was measured by reading absorbance at 595 nm using a FilterMax F5 Multimode Microplate Reader (Molecular Devices). All compounds were tested in duplicate.


$$\begin{array}{c} \%\;Parasite\;growth\;inhibition\;=\frac{x-NIC}{IC-NIC}*100 \end{array}$$ (2)

The inhibition of parasite growth was calculated using Equation 2, where x represents the absorbance measurement of the sample, IC is the absorbance of the infected control (maximum parasite growth), and NIC is the absorbance of the non-infected control (minimum parasite growth). Compounds exhibiting ≥ 60% *T. cruzi* growth inhibition and ≥ 35% Vero cell viability at the screening concentration were classified as direct hits and considered for subsequent evaluation in EC_50_ dose-response assays. For compounds exhibiting ≥ 60% *T. cruzi* growth inhibition but ≤ 35% Vero cell viability at the screening concentration, the trypanocidal assay was repeated under identical experimental conditions, but concentrations were reduced to 2 μM and 0.2 μM.

All EC_50_ dose-response experiments were performed in 96-well plates using the same experimental setup described above. Compounds were tested starting at concentrations up to 100 μM for CID-5, 50 μM for CID-6 and CID-19, 5 μM for CID-7 and 7.5 μM for CID-21 using nine serial 1:2 dilutions. Each condition was assayed with two technical replicates and three biological replicates. Each biological replicate was performed using an independant batch of parasites passaged through mice and subsequently maintained in culture. Trypanocidal activity was calculated using Equation 2 and data was imported into GraphPad Prism version 8.0.1 (GraphPad Software LLC) for analysis. EC_50_ values were calculated independently for each compound using a nonlinear regression model to generate log(dose)-response curves with a variable slope. The selectivity index (SI) was calculated for each compound as the ratio of CC_50_ to EC_50_.

### Beta-galactosidase inhibition assay

Selected compounds were tested for inhibition of β-galactosidase activity to eliminate potential false positives, i.e., compounds able to mimic true trypanocidal drugs by interfering with the reporter enzymatic assay. An extract of transgenic *T. cruzi* Tul β-gal parasites was used as the source of β-galactosidase. Transgenic parasites expressing β-galactosidase were obtained from the culture medium of infected cells, as previously described. The supernatant from a cell culture flask was centrifuged at 5,700 x g for 10 minutes and the pellet was washed twice with PBS. The resulting pellet was resuspended in 100 μl of PBS, supplemented with 0.5% NP-40 and 1 μM E-64 protease inhibitor, and incubated for 1 hour at 37 °C. After incubation, the parasite lysate was sonicated in four 5-second cycles at 5% amplitude, followed by centrifugation at 16,200 x g for 30 minutes at 4 °C. The supernatant was collected, supplemented with 20% glycerol, and stored at -80 °C until use.

Compounds were preincubated for 30 min at 37 °C with a β-galactosidase–containing lysate (1:2000 dilution) in activity buffer (PBS supplemented with 2% RPMI, 0.25% DMSO, and 0.5% NP-40, pH 7.4). The CPRG substrate was then added to a final concentration of 50 μM. Data acquisition and analysis were performed as described above. Residual β-galactosidase activity was calculated for each condition using Equation 1 and used to generate dose–response curves. Isopropyl β-D-1-thiogalactopyranoside (IPTG, 0–5 mM) served as a β-galactosidase inhibition control.

### Intracellular amastigote proliferation assay by fluorescence microscopy

Compound CID-7 was evaluated for its activity against intracellular amastigotes of *T. cruzi* using a fluorescence microscopy-based assay. This approach allows for the direct quantification of parasite burden within infected host cells through visualization of DAPI-stained nuclei and kinetoplasts. Vero cells were seeded at a density of 5 x 10^3^ cells per well in a volume of 300 μl of MEM supplemented with 10% fetal bovine serum in thin-bottom, black 96-well μ-Plates (Ibidi 89626). Cells were allowed to settle for 20 minutes and then incubated at 37 °C for 48 hours to achieve adequate adherence.

Following the initial incubation, each well was infected with 5 x 10^4^ transgenic *T. cruzi* Tul β-gal trypomastigotes in 300 μl of MEM containing 4% fetal bovine serum. The infection was allowed to proceed for 24 hours at 37 °C. After the infection period, the medium was removed and wells were washed twice with phosphate-buffered saline (PBS) to eliminate extracellular parasites. Subsequently, 300 μl of RPMI medium supplemented with 0.5% DMSO containing the test compound was added to each well.  CID-7 was evaluated at two concentrations, 1$\times {\text{IC}}_{50}$ and 3$\times {\text{IC}}_{50}$, with each condition assayed in duplicate. Benznidazole (BNZ) was included as a reference control at the same concentrations (1$\times {\text{IC}}_{50}$ and 3$\times {\text{IC}}_{50}$), while untreated wells containing 0.5% DMSO served as negative controls.

After 72 hours of treatment, the plate was fixed with 1% paraformaldehyde for 20 minutes at room temperature. Following fixation, nuclei were stained with DAPI in mounting medium (Ibidi 50011). Image acquisition was performed using an Axio Observer 7 (Zeiss) microscope, capturing 25 images per well at 40× magnification.

## Results

### Data exploration and filtering strategy

To identify compounds with potential for repurposing against *T. cruzi*, a query was designed to retrieve molecules known to be active against a validated target in another organism, provided that an orthologous gene exists in *T. cruzi* and that the compound has no recorded activity against *T. cruzi*, *T. brucei*, or *L. major*. This strategy enables the prioritization of candidates that may act on *T. cruzi* through conserved biological targets but have not yet been evaluated in that specific context. This query was called the “TDR Targets Query Dataset” (Step 0) and contained a subset of 327 genes. From these protein coding genes, we then extracted the entire set of bioactive compounds associated with them by TDR Targets (approximately 180,000).

To bridge the gap between associations derived from the chemogenomic network and experimental feasibility, the network derived putative interactions set was subjected to a multi-stage filtering process using a set of Python scripts (available in a Jupyter Notebook). As the first step (Step 1), a novelty filter was applied to exclude compounds already present in DrugBank [60] or previously tested against trypanosomatids, leaving a refined set of 178,144 molecules. A second step (Step 2) was added recognizing that commercial accessibility is a critical bottleneck in neglected disease research. We programmatically validated the entire set via the MolPort API, finding that only ~ 15% (29,880 compounds) had a similar compound available for purchase at the time of this assessment and that 28,802 were an exact match with the ones prioritized in this work.

The third step (Step 3) narrowed the scope to 44 core metabolic genes, based on their involvement in energy and amino acid metabolism, associated with 4,041 commercially available compounds. This *ad hoc* filtering step was based on evidence indicating that carbon metabolism is notably enhanced in cells infected with amastigotes [61], as well as the role of certain amino acids (and intermediate metabolites) in parasite survival during infection [62].

**Fig 1:**
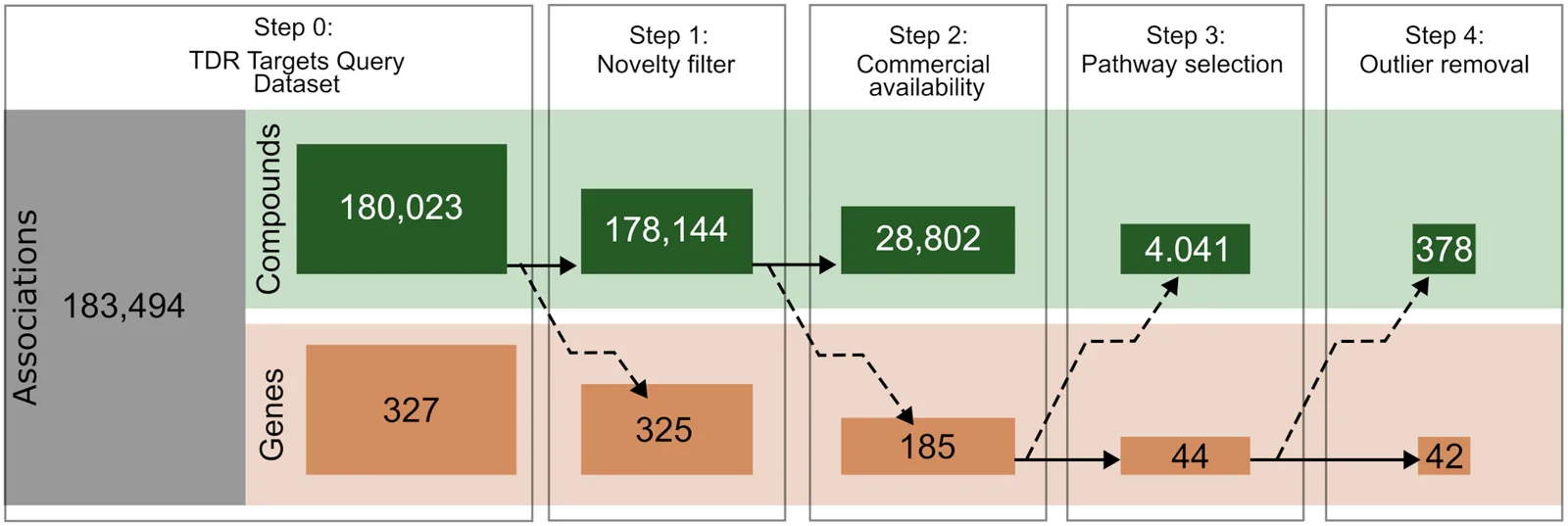
Compound and target gene selection workflow. The initial dataset of 180,023 compounds and 327 target genes was sequentially filtered by novelty and commercial availability, resulting in 28,802 compounds. Pathway-based selection reduced the dataset to 4,041 compounds and 44 genes, followed by outlier removal yielding a final set of 378 compounds and 42 genes.

The fourth and final step (Step 4) of the pipeline focused on structural clustering and outlier removal to ensure the selection of high-quality chemical leads. This systematic curation yielded a final set of 378 compounds associated with 42 genes. The list of genes and the number of compounds associated with each one is shown in S1 Table. Figure 1 shows the order in which the datasets were combined and the number of genes and compounds obtained in each case.

### Construction of screening libraries

Based on the cleaned clustered data, a substructure search was performed for sets of functional groups reported in the literature as potentially trypanocidal (see Methods). The compound candidates were partitioned into 16 distinct scaffold-based libraries. Each scaffold was selected because it has been reported in compounds with trypanocidal activity. The complete list of scaffolds can be found in Table 2. Using this information, we partitioned our dataset into focused libraries: each library was characterized by containing compounds featuring one or more of these functional groups. The libraries were not exclusive (a molecule may belong to one or more libraries). This sub-grouping aimed to facilitate the final exploration of the libraries. All the libraries are available through Github.

**Fig 2:**
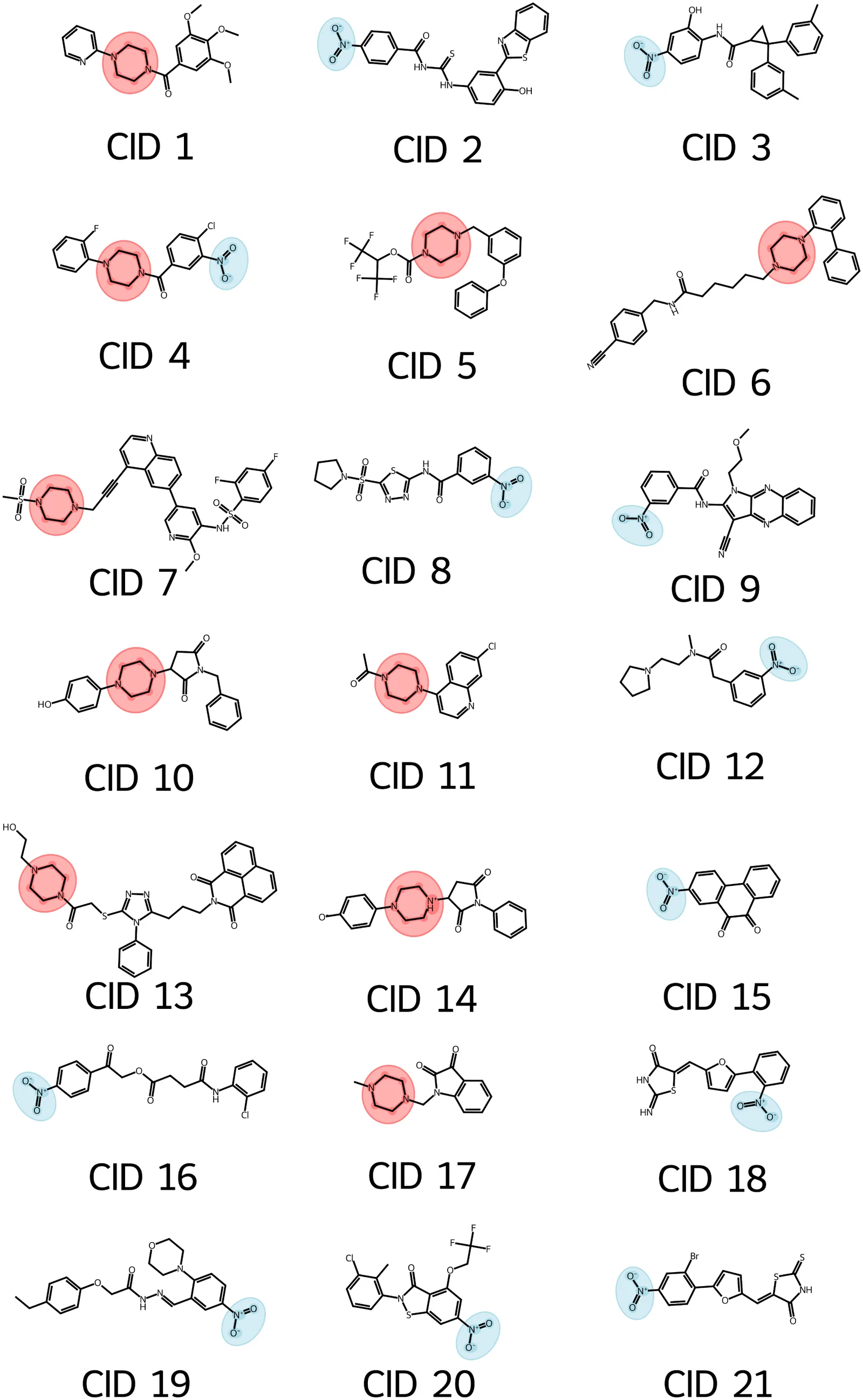
Selected compounds for testing. Purchased compounds are arranged in a grid layout, with each structure labeled by a Compound Identifier (CID). The core scaffolds that define the chemical series explored in this work are schematized and color-coded for clarity: the nitro scaffold is highlighted in light blue, and the piperazine scaffold is highlighted in coral.

For experimental validation we selected the nitro and piperazine chemical libraries guided by two distinct yet complementary rationales. Nitro-containing compounds were prioritized due to their well-documented history as reactive (class-specific) pharmacophores with potent anti-*Trypanosoma cruzi* activity [49]. In contrast, piperazines were selected not for intrinsic reactivity, but for their versatility as structural scaffolds and linkers [63]. The piperazine scaffold provides conformational flexibility and multiple points for chemical diversification, enabling the presentation of a wide range of appended functional groups with the potential to engage diverse biological targets (generalistic) [64,65]. Together, the inclusion of these two libraries reflects a strategy to incorporate chemical sets with different origins and mechanistic rationales: one based on known parasite-specific chemical reactivity and the other on scaffold-driven structural versatility and exploratory potential. From these two libraries we selected 21 compounds to test *in vitro* (Fig 2). The selection reflected mainly short term availability and price affordability at the time of purchase.

### Experimental validation: Primary screening

**Fig 3:**
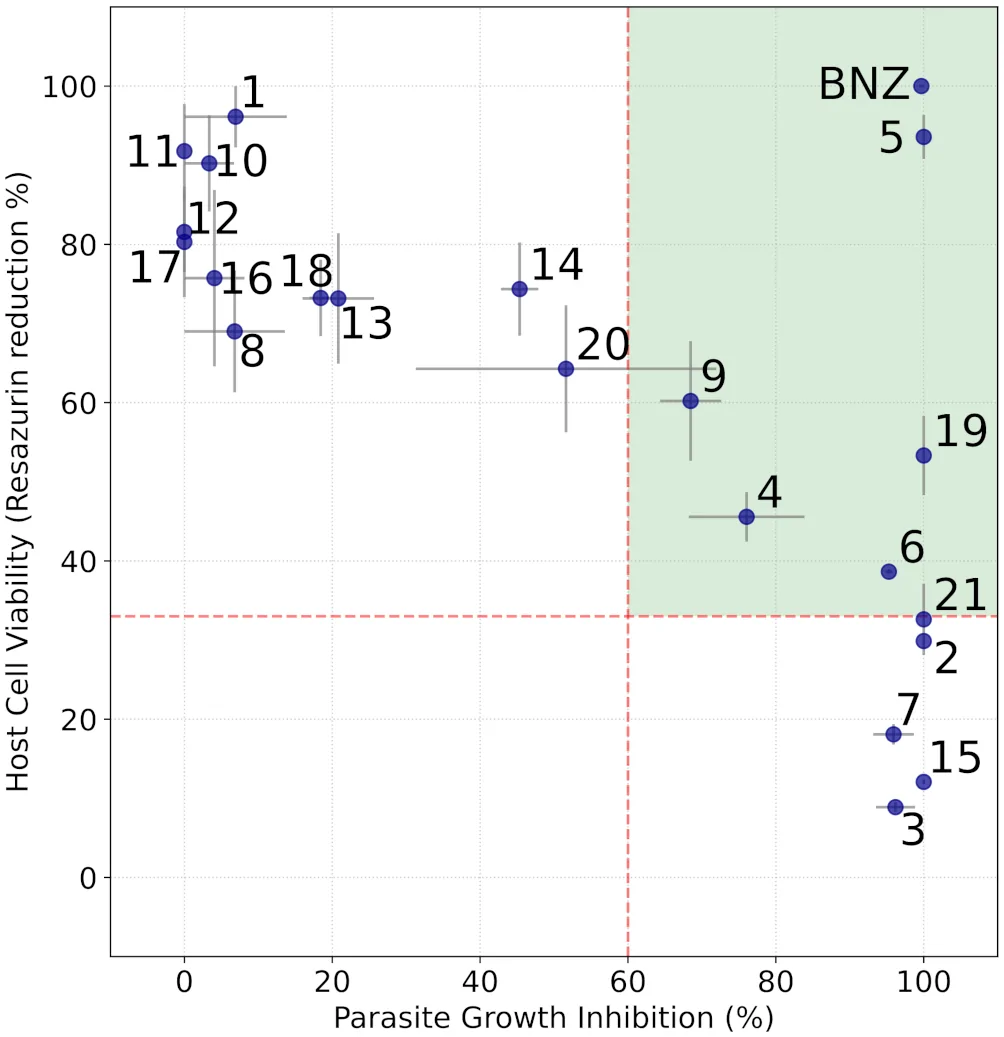
Trypanocidal activity vs. cytotoxicity plot from primary screening. Each compound was tested in duplicated at a fixed dose of 20 μM. Results from the β-galactosidase activity and resazurin reduction assays are shown for each compound. The horizontal axis represents the percentage of *T. cruzi* amastigote growth inhibition based on β-gal activity measured with the CPRG substrate. The vertical axis shows cell viability as a function of the percentage of resazurin reduction at a fixed time point: the higher the percentage, the higher the cellular metabolism, and thus the lower the compound’s cytotoxicity. Horizontal and vertical bars for each point represent measurement errors, expressed as the standard deviation between replicates and their average. Compounds of interest appear in the upper right quadrant (high inhibition of parasite growth, high cellular viability) and are labeled as ‘CID-number’, along with BNZ (Benznidazole), which was used as a positive control. Compounds in the lower right quadrant were also selected for retesting at lower concentrations. Molecules in the left quadrants were considered negative.

In a primary screening, the 21 selected compounds were tested *in vitro* to assess their cytotoxicity and trypanocidal activity at a fixed concentration of 20 μM (see Methods). Results from primary screening are summarized in Fig 3.

For this assay, thresholds were set at 35% for cytotoxicity and 60% for growth inhibition. The growth inhibition threshold was set at 60%, consistent with standard practices in phenotypic screening for *Trypanosoma cruzi*, where this cutoff is commonly used to identify compounds with moderate to robust anti-parasitic activity [66]. For cytotoxicity, a conventional threshold of 60% host cell viability is typically employed to define overt toxicity. However, applying this stringent cutoff in our campaign resulted in the exclusion of all compounds from further analysis, leaving only one viable hit for downstream evaluation. Therefore, following established HTS strategies where hit thresholds can be tailored to follow-up capacity, the cytotoxicity threshold was relaxed to 35% [67].

While 11 compounds showed only moderate or no antiparasitic activity (inhibition% < 60%), other 11 compounds, including the reference drug BNZ, significantly inhibited parasite growth at 20 μM on the primary screening. Of them, CID-2, CID-3, CID-5, CID-6, CID-7, CID-15, CID-19, and CID-21, completely arrested parasite growth (% inhibition> 90%) at this concentration, standing out as the most promising candidates. Contrary to their uniform antiparasitic activity, these compounds displayed highly disparate toxicity on Vero cells. Five compounds (CID-2, CID-3, CID-7, CID-15, and CID-21) were highly toxic (cell viability % < 35%) at 20 μM, and three (CID-5, CID-6, and CID-19) exhibited moderate to low cytotoxicity. Of note, CID-5 showed the most favourable activity/cytotoxicity profile among the investigated candidates, with almost maximal anti-*T. cruzi* activity and negligible cytotoxicity on host cells.

To explore for differential effects on parasite and cell viability at other concentration ranges, the five compounds showing the highest cytotoxicity were re-tested at lower doses of 2 μM and 0.2 μM (S1 Fig). CID-2 failed to reproduce the bioactivity observed in the previous screening round and CID-15 showed similar effects on both parasites and Vero cells, indicating indiscriminate toxicity. Thus, both compounds were discarded. On the contrary, CID-3, CID-7, and CID-21 exhibited highly selective bioactivity profiles at 2 μM. At this concentration, these compounds inhibited parasite growth almost completely (Inhibition % > 95%) while demonstrating high cell viability (70%-85%), indicative of acceptable selectivity ratios. All the compounds showed only negligible bioactivity at the lowest concentration tested (0.2 μM).

In summary, after primary screening, 8 compounds (CID-3, CID-4, CID-5, CID-6, CID-7, CID-9, CID-19, and CID-21) remained as viable candidates, as they showed reproducible and selective antiparasitic activity (Fig 3). The top five hits CID-5, CID-6, CID-7, CID-19, and CID-21 were prioritized for further dose-response analysis in secondary screening.

### Dose-response analysis and enzymatic triaging of top hits

**Fig 4:**
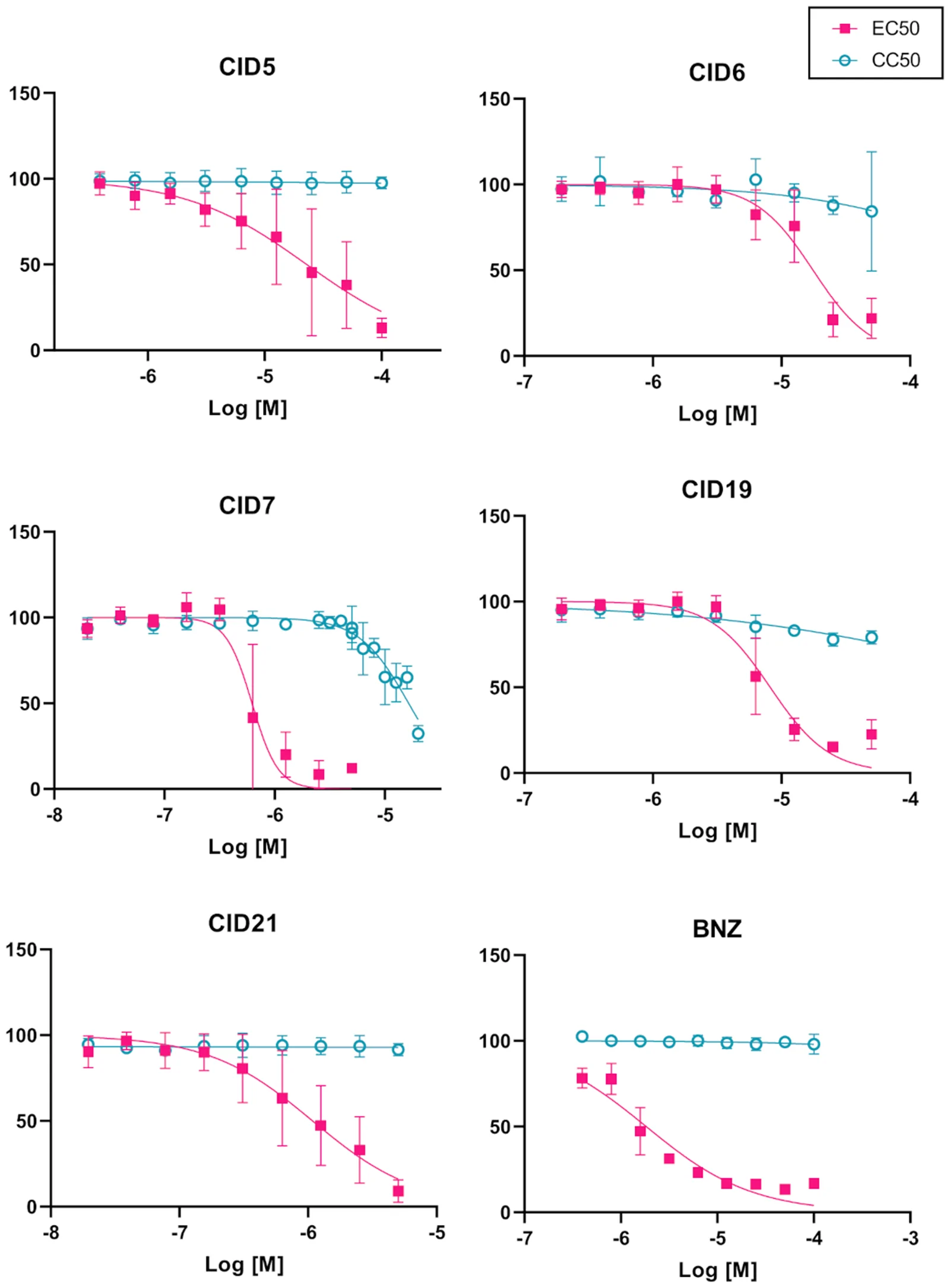
Dose-response curves for the estimation of CC_50_ and EC_50_ in confirmatory screening. Selected hits from primary screening were re-tested across a range of concentrations to generate dose-response curves, from which EC_50_ and CC_50_ were estimated. Curves for the antiparasitic and cytotoxic activity of each compound are indicated in fuchsia and cyan, respectively. Each point represents the mean and SD of the biological replicates.

Dose-response assays were performed to estimate half-maximal effective (EC_50_) and cytotoxic (CC_50_) concentrations of each compound under standardized conditions. As expected from primary screening, the analysis revealed marked differences among compounds in both antiparasitic potency and selectivity. The most potent and selective molecule, CID-7, exhibited a submicromolar EC_50_ ($6.22\times 10^{-7}$ M) together with the highest selectivity index (SI = 26.33), indicating a favorable therapeutic window. CID-21 and CID-19 also showed antiparasitic potencies in the low micromolar range, with EC_50_ values of $1.08\times 10^{-6}\text{ M}$ and $8.17\times 10^{-6}\text{ M}$, respectively, and moderate selectivity. Despite its promising activity against *T. cruzi* in primary screening, CID-5 displayed an EC_50_ value of $2.29\times 10^{-5}\text{ M}$ and a modest selectivity index. Among the selected hits, CID-6 was the least selective candidate. Although no signs of cytotoxicity were detected in dose-response analysis, this compound showed only a modest antiparasitic potency (EC_50_ = $1.76\times 10^{-5}\text{ M}$), resulting in a poor selectivity index (SI > 2.84). Figure 4 and Table 3 summarizes dose–response curves and calculated parameters for these compounds.

**Table 3:** Compound activity data against *T. cruzi.*

CID	Library	EC_50_ (M)	[95% CI]	CC_50_ (M)	Selectivity index (SI)	IC_50_ (M) β-gal inhibition
5	Piperazine	$2.29\times 10^{-5}$	[$1.57\times 10^{-5}$ – $3.68\times 10^{-5}$]	> $1\times 10^{-4}$	> 4.37	> $1\times 10^{-4}$
6	Piperazine	$1.76\times 10^{-5}$	[$1.51\times 10^{-5}$ – $2.05\times 10^{-5}$]	> $5\times 10^{-5}$	> 2.84	> $1\times 10^{-4}$
7	Piperazine	$6.22\times 10^{-7}$	[$5.36\times 10^{-7}$ – $7.63\times 10^{-7}$]	$1.64\times 10^{-5}$	26.33	> $1\times 10^{-4}$
19	Nitro	$8.17\times 10^{-6}$	[$7.04\times 10^{-6}$ – $9.57\times 10^{-6}$]	> $5\times 10^{-5}$	> 6.12	> $1\times 10^{-4}$
21	Nitro	$1.08\times 10^{-6}$	[$8.34\times 10^{-7}$ – $1.41\times 10^{-6}$]	> $5\times 10^{-6}$	> 4.61	> $1\times 10^{-4}$
BNZ	–	$1.83\times 10^{-6}$	[$1.45\times 10^{-6}$ – $2.29\times 10^{-6}$]	> $1\times 10^{-4}$	> 54.59	N/A

Finally, the investigated compounds were tested in an enzymatic triage to discard candidates able to interfere with the reporter methodology (i.e., β-galactosidase inhibitors or highly coloured compounds) used in the screening assay. In the range of concentrations tested, none of the investigated compounds showed detectable β-galactosidase inhibition (S3 Fig), indicating that all the hits genuinely reduced parasite burden in culture. To further confirm the anti-*T. cruzi* activity of the top hit CID-7, this compound was evaluated in an independent fluorescence microscopy-based intracellular amastigote proliferation assay (S4 Fig). As shown in S4 Fig, CID-7 markedly reduced the parasite burden in a concentration-dependent manner, confirming the results obtained in the primary screening. Overall, our results validate CID-5, CID-7, CID-19, and CID-21 as *bona fide* hits with selective anti-*T.cruzi* activity with potential as starting point for further optimization and confirm the libraries generated from TDR Targets as enriched sources of attractive candidates.

## Discussion

Despite decades of intense research, Chagas disease remains the most important parasitic disease in the Americas, with the only two drugs in clinical use displaying suboptimal efficacy and safety profiles. To cope with the urgent need for new and safer therapeutic options for this disease, in this work we developed an integrative workflow to prioritize chemical scaffolds with potential activity against *T. cruzi*. Using the TDR Targets Database [19], we applied an *ad hoc* filtering strategy to assemble 16 scaffold-specific libraries comprising 378 drug-like compounds, which are freely available to the scientific community. Experimental validation of 21 compounds from two libraries confirmed four novel and promising anti-*T. cruzi* hits. The bioinformatics and chemoinformatics pipeline was implemented in python and is available as a Jupyter notebook [68] for direct use or modification by users and can be used to further analyze subsets of data obtained from TDR Targets (or any other similar database).

### New set of compounds with validated activity against *T. cruzi*

From the initial 21 candidates tested in primary assays, eight showed reproducible anti-*T.cruzi* activity at fixed concentrations. The top 5 compounds were assessed in a secondary screening, and 4 demonstrated suitable selectivity over Vero cells in dose-response analysis. Notably, the most potent and selective candidate identified in this work, CID-7, matches two commonly used criteria determining compound progression in phenotypic development pathways: EC_50_ ≤1 μM and SI ≥ 25 [69]. Importantly, its identification resulted from our decision to relax the cytotoxicity cut-off to 35%. This decision not only proved correct but also demonstrates that preliminary activity/toxicity assessments at fixed doses should not be considered “hard” thresholds but only references for early candidate prioritization.

**Fig 5:**
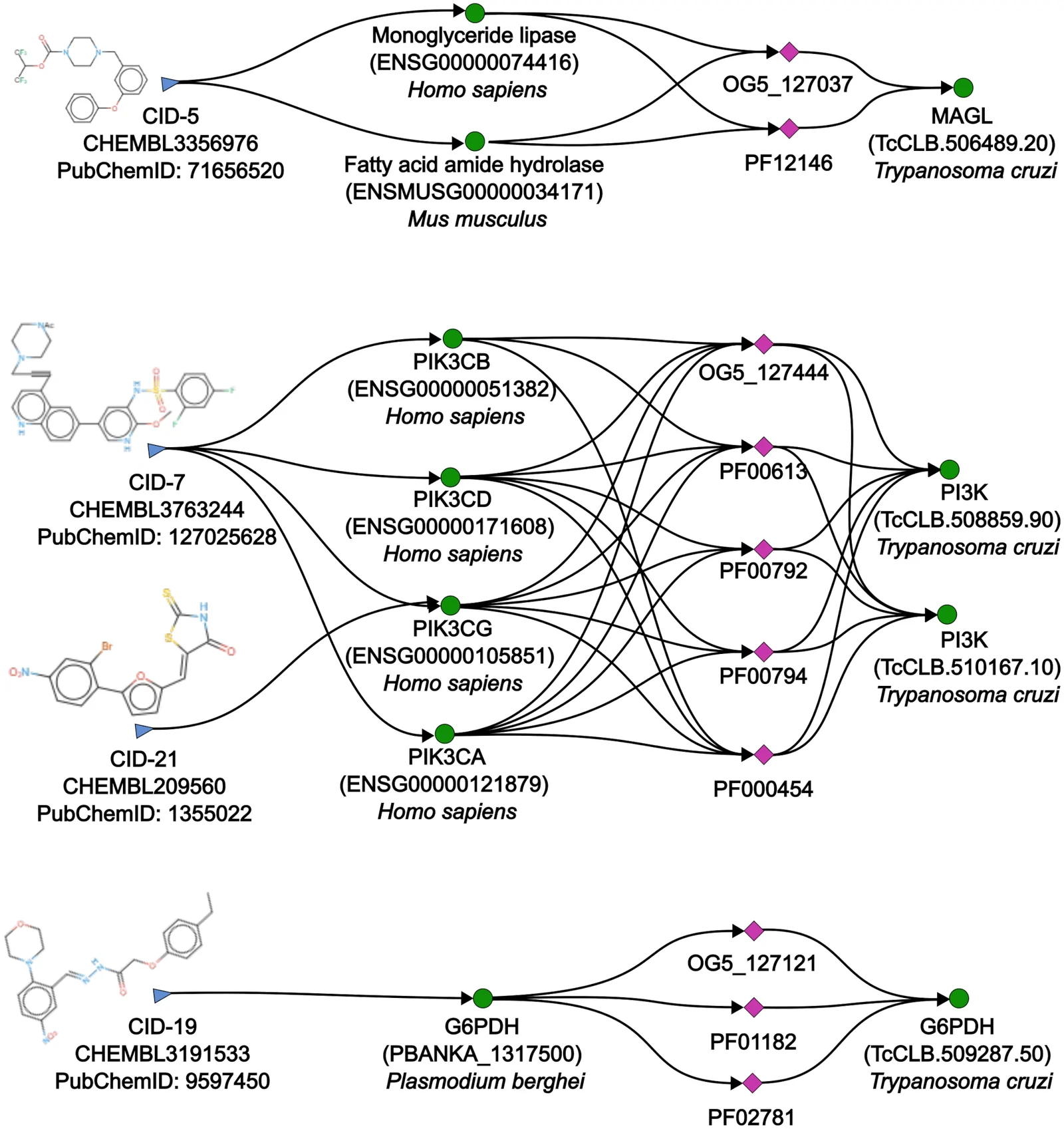
Top 4 hits from the primary screening, extracted subgraphs from TDR Targets, and putative targets. Chemical structures of the top screening hits and the corresponding subgraph retrieved from TDR Targets for each compound. In the graph, blue nodes represent compounds, green nodes represent protein targets, and magenta nodes correspond to functional annotations. The green nodes on the right side of each graph indicate the putative *T. cruzi* targets.

We explored the trypanocidal activity of just over 5% of the prioritized molecules, suggesting that there is still significant undiscovered potential in the results of this prioritization. Regarding the novel active compound found, we went back to the TDR Targets web app to explore resultant hits, and visualize the chemical neighborhood around selected compounds. The putative protein targets are shown in Fig 5. S5 Fig shows the subgraphs for candidate compounds selected for experimental validation.

A nitro-containing compound library was chosen due to the validated, though complex, pharmacophore shared by the frontline drugs BNZ and NFX. While this nitro group is associated with potent anti-parasitic activity [49,70], it can also contribute to off-target reactivity, metabolic toxicity, and genotoxic potential, which are concerns for the existing therapeutics [71,72]. Among our hits, CID-4 and CID-9 contained nitro groups and displayed moderate cytotoxicity alongside trypanocidal activity, consistent with this challenging profile. However, our pipeline also identified CID-21, a nitro-containing hit, which displays a suitable activity/toxicity profile. This suggests that not all nitro-aromatics share the same promiscuous toxicological profile, and that structural context is critical. Further development of any nitro-containing hit from this library would necessitate rigorous early-stage ADMET profiling to assess whether it offers a genuinely improved therapeutic index over current nitro-heterocyclic drugs.

The decision to experimentally screen a piperazine-based library was predicated on its established role as a privileged, flexible scaffold in medicinal chemistry, valued for its ability to serve as a conformational spacer and present diverse pharmacophores to biological targets. In practice, the piperazine scaffold acts as a molecular dispenser, capable of presenting a diverse array of pharmacophores to biological targets by projecting them into distinct regions of a binding site [63]. The inherent flexibility of the piperazine ring allows it to adopt different conformations [64], meaning that depending on the substituents attached, the molecule can change its conformations (e.g., chair, half-chair, boat, twist-boat), and thus may fit the unique contours of protein pockets [65]. This exploratory, scaffold-driven strategy proved fruitful. Among our hits, the piperazine scaffold was incorporated into chemically distinct architectures, such as in CID-5 and CID-7. Notably, these piperazine-containing hits were computationally associated with different putative target classes (see next section). This divergence supports the core premise of our selection: the piperazine scaffold itself did not confer a single, predetermined mechanism of action but rather facilitated the discovery of structurally novel compounds whose activity likely stems from their unique, appended functional groups. The success of this series validates the inclusion of such ‘framework’ scaffolds in prioritization pipelines, as they can efficiently sample disparate regions of chemical and target space, moving beyond analogues of known inhibitors to uncover genuinely novel chemotypes.

Although one might expect a correlation between library membership and mechanism of action, it is important to note that, at this point, it is impossible to determine whether these scaffolds are actually responsible for any observed trypanocidal activity. Two alternative hypotheses can be proposed about their action: they either inhibit a target or pathway in the host cell that is necessary for the replication of the parasite, or they inhibit a target in the parasite itself. This should be investigated in future work.

Regarding the proposed candidate targets and the observed selectivity (EC_50_ vs CC_50_), it should be considered that we only validated a small number of compounds for practical and economical reasons. The TDR Targets database integrates genomic, functional, and chemical data from both human pathogens and model organisms, using orthology relationships to infer bioactivity evidence from well-studied species (human, mouse, rat, etc.) to understudied neglected disease pathogens [19]. Hence while it provides a proof of concept that the prioritization strategy can yield active compounds, it also serves to highlight the importance of carefully assessing the selectivity of all the candidates, and other analogs when undertaking the experimental validation of the other ~ 300 compounds from the 14 remaining libraries produced in this work. As an example it is worth noting that the strategy relies on repositioning of compounds to *T. cruzi* from often complex topologies of the network subgraphs that connect targets, and compounds (number of nodes, multiplicity of interconnections, co-occurrence of positive and negative bioactivity associations, see Fig 5). Hence there is ample room for improving these hit compounds further through exploration of the chemical space around them, and when validating their respective targets and mechanisms of action.

### Putative Targets of the validated hits

**Fig 6:**
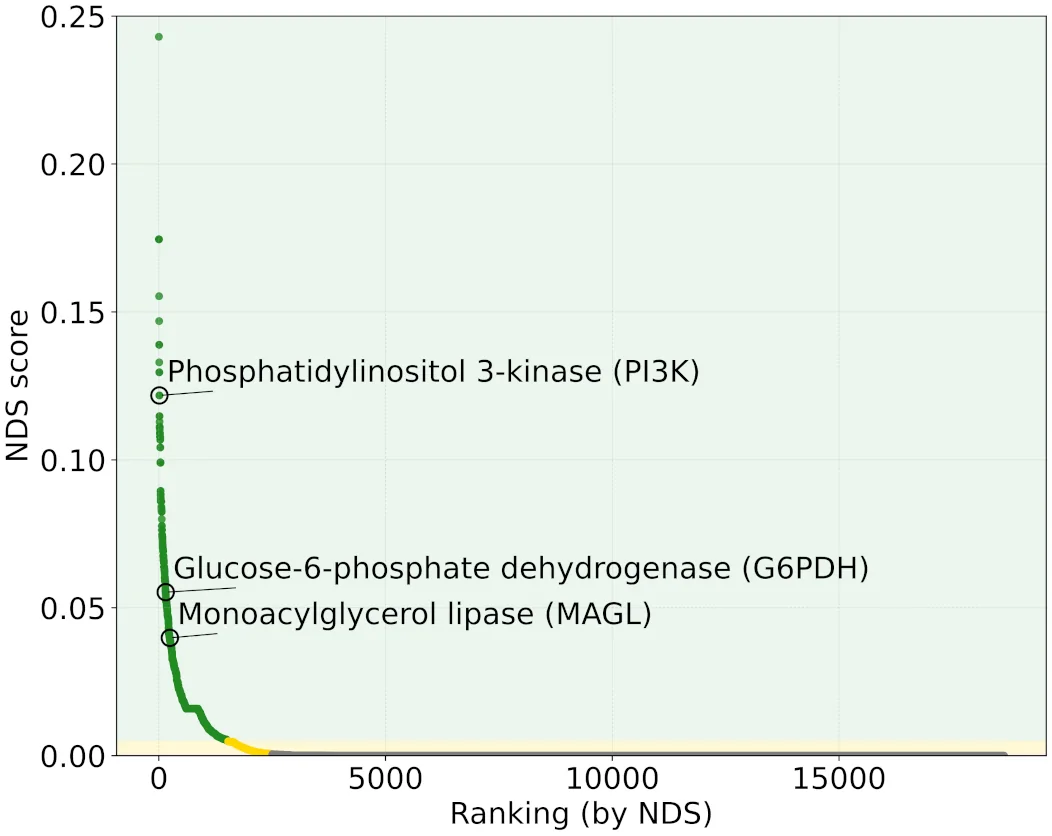
Ranking of the *Trypanosoma cruzi* proteome based on the Network Druggability Scores (NDS). Full ranking of all protein-coding genes in *T. cruzi* based on their NDS. The x-axis represents genes ranked in descending order of their score, while the y-axis shows the quantitative NDS value. This metric reflects the probability of a protein being a successful drug target, derived from its connectivity within a multilayer chemogenomic network and the enrichment of bioactive compounds in its functional neighborhood. High-confidence candidates are located at the far left of the ranking, where scores are highest. The shaded background delimits statistical confidence zones: the green zone (highest confidence - DG 5 and 4), the yellow zone (DG 3), and the red zone (low-confidence targets below baseline noise - DG 2 and 1). The proposed targets for active compounds are identified by labels.

Concerning the proposed targets of the 4 prioritized compounds, CID-5 appears as a possible inhibitor of a monoacylglycerol lipase, MAGL (TcCLB.506489.20). Interestingly, CID-7 and CID-21, which belong to the piperazine and nitro libraries, respectively, and showed a low Tanimoto similarity index (0.387), were both associated with the same *T. cruzi* target: phosphatidylinositol 3-kinase, PI3K (TcCLB.510167.10). Finally, CID-19 and CID-6 both appeared as putative inhibitors of glucose-6-phosphate dehydrogenase, G6PDH (TcCLB.509287.50). All three targets have a high NDS (Fig 6).

It is important to note that the drug-target associations obtained by the network model in this work are fallible, so it cannot be stated with certainty that the targets of these molecules are unequivocally the ones indicated. In the discussion that follows, we worked under the hypothesis that the proposed targets are a sufficient approximation for making decisions about which of the molecules (and their respective putative targets) are worth continuing to study.

G6PDH is a widely studied target in *T. cruzi* [73,74]. In addition to participating in the glycolytic pathway for energy generation, it is believed to have a defensive/regulatory role against oxidative stress caused by the environment [75,76], which positions this enzyme as a possible secondary target for combination therapies with BNZ or NFX.

Phosphatidylinositol 3-kinases (PI3Ks) are members of a kinase superfamily involved in signal transduction that drives various complex cellular processes, such as cell growth, proliferation, differentiation, motility, and trafficking, among others [77,78]. In mammals, this family is divided into three functional classes (I, II, and III) based on their structural organization and substrate preference [77]. However, in trypanosomatids, only Class I and Class III orthologs are present, as Class II PI3Ks appear to be exclusive to multicellular eukaryotes and have not been reported in these unicellular parasites [79]. To date, the Class III member TcVps34 has been characterized in *Trypanosoma cruzi*, playing a vital role in osmoregulation and receptor-mediated endocytosis [34]. Due to their essential nature, PI3Ks have been evaluated as potential therapeutic targets [80]. In phenotypic screenings with known human PI3K inhitors against trypanosomatids, some compounds like NVP-BEZ235 (Dactolisib - an anticancer drug that inhibits PI3K and mTOR in mammals and inhibits the cell growth of cancer cells) showed sub-nanomolar potency [81], and its effectiveness was demonstrated both *in vitro* and *in vivo* for *L. donovani* and *T. brucei* [82]. This precedent provides fertile ground supporting the repositioning of human PI3K and mTOR inhibitors to Chagas disease.

We assessed the molecular similarity of CID‑7 and CID‑21 against a panel of reference PI3K inhibitors [77,81,83] using 2D and 3D similarity metrics (S3 Table). Both compounds showed generally low to moderate 2D similarity across all references indicating that their core scaffolds are distinct from those of the reference inhibitors. However, CID‑7 exhibited notable 3D similarity with two inhibitors, PIK‑90 and Torin‑2, both are known to bind the ATP‑binding pocket of class I PI3Ks. Importantly, LY294002 and PIK‑90 have been previously shown to inhibit the recombinant class I PI3K from *T. cruzi* (TcPI3K), the only class I isoform characterized in this parasite so far. The higher 3D overlap of CID‑7 with these inhibitors suggests that it may adopt a similar conformation within the active site of TcPI3K. The molecules found in this work and potentially associated with this enzyme are not structurally related to each other, or to Dactolisib, or other molecules previously studied in trypanosomatids, thus offering two possible new families of inhibitors for this enzyme. Importantly, although Dactolisib failed in clinical trials due to poor tolerability and lack of efficacy [84], our compounds are structurally distinct and may therefore overcome these limitations. This structural novelty positions them as promising leads for further optimization.

Finally, monoacylglycerol lipase (MAGL, EC 3.1.1.23) is the enzyme that catalyzes the degradation of monoglycerides (intermediate lipids derived from phospholipid degradation) into glycerol and fatty acids, with arachidonic acid as the main chemical species, due to its abundance but also due to its relevance in various signaling cascades that regulate synaptic function and inflammation in mammals [85]. In trypanosomatids, its function is not clear, but there is evidence that it may act as a virulence factor, possibly modulating the host’s innate immunity [86,87]. To date, there are no reports of MAGL inhibition in trypanosomatids; if interaction is confirmed by biochemical assays, CID-5 would be the first reported compound capable of inhibiting this enzyme in *T. cruzi*. All enzymes involved in eicosanoid metabolism are extensively studied in mammals, and there are currently multiple inhibitors for the entire pathway and for MAGL in particular, which presents a rich scenario for both direct drug repositioning and for the biological characterization of the enzyme and its study as a potential therapeutic target.

## Conclusion

As shown in this work, it is possible to create screening libraries using TDR Targets, as it enables not only the prioritization of targets but also the expansion of compound series through both existing and inferred relationships between entities within the network. This means that, for any given target of interest, potential inhibitors can be identified based on evidence obtained for homologous proteins in other organisms, as well as structurally similar compounds to a known inhibitor, based on proximity and relevance calculations within the network.

Through the developed pipeline, several valuable candidates were identified and experimentally validated. Although only two from the 16 generated libraries were explored, it is encouraging that the number and quality of the hits validated here are similar to those obtained from similar computational approaches [88–90].

An additional possible advantage of this type of approach is that the prioritization of molecules comes with their putative target. Although there are no guarantees that the recovered target is exactly the one suggested by TDR Targets, the association is highly probable. This provides an actionable starting point for molecules with an interesting trypanocidal profile, and also an extra piece of information to re-prioritize if it becomes necessary to further narrow down the number of molecules. In this work, putative inhibitors were obtained for G6PDH, MAGL, and PI3K. Of these, the MAGL enzyme is possibly the most interesting target that emerged from this study. At the same time, orthologs of this enzyme are extensively studied in model [91–93] and pathogenic [94–97] organisms, which offers a large repertoire of available molecules for repositioning and biochemical assays to adapt.

Throughout this work, the potential of using integrative chemogenomics for drug discovery and repositioning in neglected diseases has been demonstrated, with a special focus on Chagas disease. Our results serve as the foundation for new lines of research focused on the validation of putative targets, the expansion of chemical series based on the identified hits, and the elucidation of the possible mechanisms of action involved in their trypanocidal activity.

## Supporting information

S1 FigDose-response re-test of selected hits against *Trypanosoma cruzi* and Vero cells.Scatter plots representing the relation between *T. cruzi* growth inhibition (%) and Vero cell viability (%) at three different concentrations: (A) 20 μM, (B) 2 μM, and (C) 0.2 μM. Data points represent the mean and standard deviation (SD) of two independent biological replicates. The horizontal dashed red line indicates the cytotoxicity threshold (35% viability), and the vertical dashed red line indicates the anti-parasitic activity threshold (60% inhibition). CN: Negative Control (Culture medium); BNZ: Benznidazole (Positive control); DMSO: Dimethyl sulfoxide (Vehicle control); 2, 3, 7, 15, 21: CID numbers of the re-tested compounds.(TIFF)

S2 Fig Dose–response curves from independent biological replicates. Dose–response analyses for CID-5, CID-6, CID-7, CID-19 and CID-21. Each curve represents an independent biological replicate, with symbols corresponding to individual experiments. Data points are shown as mean values of technical replicates. Fuchsia curves correspond to EC50 determinations for trypanocidal activity, whereas cyan curves correspond to CC_50_ determinations for cytotoxicity in Vero cells. Curves were fitted using nonlinear regression.
(TIFF)

S3 FigResults of the β-galactosidase inhibition assays.Active compounds were evaluated for β-galactosidase inhibition using parasite lysates. IPTG was included as a positive control. In the range of concentrations tested, none of the compounds exhibited inhibitory activity against the reporter enzyme. Each point represents the result of a single biological replicate.(TIFF)

S4 FigFluorescence microscopy analysis of infected cultures treated with the investigational compound CID-7.Vero cells (5 x 10^3^ per well) were seeded in thin-bottom, black 96 well μ-Plates (Ibidi 89626) and infected with Tul β-Gal trypomastigotes ( 5 x 10^4^ per well) as in previous experiments. Cultures were incubated with the compound or vehicle at the indicated concentrations for 72 h, fixed, and stained with DAPI in mounting medium (Ibidi 50011). Each condition was assayed in duplicate. Twenty-five images/well were obtained at 40x magnification in an Axio Observer 7 (Zeiss) microscope. Top panel (A-D): Representative DAPI images of infected cultures treated with drug vehicle (DMSO). Middle panel: Representative DAPI images of infected cultures treated with CID7 at 1xEC_50_ (E-H) or 3xEC_50_ (I-L). Bottom panel: Representative DAPI images of infected cultures treated with the reference drug Benznidazole at 1xEC_50_ (M-P) or 3xEC_50_ (Q-T).(TIFF)

S5 Fig Drug-target subgraphs for candidate compounds selected for experimental validation. Schematic representation of the subgraphs obtained for each drug-target pair within the dataset. Molecules acquired for experimental validation are distinctively labeled: compounds from the nitro library are shown in red, while those from the piperazine library are shown in green, each accompanied by its corresponding TDR Targets platform numerical identifier. Additionally, 2D chemical structures are provided to illustrate the structural diversity explored in this study. Compounds marked with a star indicate those that yielded positive results in the primary screening (see experimental validation section). Each gray node represents one or more protein targets or intermediate molecular entities within the interaction network.
(TIFF)

S1 File Library Preparation Notebook. A Jupyter interactive Python notebook used for the preparation and refinement of chemical libraries. The notebook includes annotated code and multiple visualizations that document each step of the workflow, from data integration and filtering to library construction. The notebook is publicly available at this Github repository: https://github.com/trypanosomatics/TDR-screening in the network-based-library directory.
(IPYNB)

S1 Table Genes prioritized in this study. List of the genes prioritized in this study, along with their respective *T. cruzi* CL-Brener genome locus identifiers, putative functions, druggability groups (DG), and number of compounds.
(XLSX)

S2 Table Compounds tested in this study. List of the 21 compounds experimentally tested in this study, along with their respective sources (supplier), identifiers, SMILES strings, IUPAC names and the Classyfire ontology chemical class.
(XLSX)

S3 Table 2D and 3D PI3K Ligand comparison. Summary of computed 2D and 3D similarity metrics between the identified compounds (CID-7, CID-21) and reference PI3K inhibitors.
(XLSX)
